# Protection against Virulent Infectious Bronchitis Virus Challenge Conferred by a Recombinant Baculovirus Co-Expressing S1 and N Proteins

**DOI:** 10.3390/v10070347

**Published:** 2018-06-27

**Authors:** Yuan Yuan, Zhi-Peng Zhang, Yi-Ning He, Wen-Sheng Fan, Zhi-Hua Dong, Li-Hua Zhang, Xin-Kuan Sun, Li-Li Song, Tian-Chao Wei, Mei-Lan Mo, Ping Wei

**Affiliations:** College of Animal Science and Technology, Guangxi University, Nanning 530004, China; yy05030417@126.com (Y.Y.); Zhangzhipengbest@163.com (Z.-P.Z.); heyiningf@foxmail.com (Y.-N.H.); 2003424318@163.com (W.-S.F.); dongzhihua0818@163.com (Z.-H.D.); lihuazhang1990@163.com (L.-H.Z.); sunxinkuan@163.com (X.-K.S.); sunnylily818@163.com (L.-L.S.); tcwei88@126.com (T.-C.W.)

**Keywords:** infectious bronchitis virus, protection, co-expressing, subunit vaccine, challenge

## Abstract

Avian infectious bronchitis virus (IBV) is the causative agent of infectious bronchitis, which results in considerable economic losses. It is imperative to develop safe and efficient candidate vaccines to control IBV infection. In the current study, recombinant baculoviruses co-expressing the S1 and N proteins and mono-expressing S1 or N proteins of the GX-YL5 strain of IBV were constructed and prepared into subunit vaccines rHBM-S1-N, rHBM-S1 and rHBM-N. The levels of immune protection of these subunit vaccines were evaluated by inoculating specific pathogen-free (SPF) chickens at 14 days of age, giving them a booster with the same dose 14 days later and challenging them with a virulent GX-YL5 strain of IBV 14 days post-booster (dpb). The commercial vaccine strain H120 was used as a control. The IBV-specific antibody levels, as well as the percentages of CD4+ and CD8+ T lymphocytes, were detected within 28 days post-vaccination (dpv). The morbidity, mortality and re-isolation of the virus from the tracheas and kidneys of challenged birds were evaluated at five days post-challenge (dpc). The results showed that the IBV-specific antibody levels and the percentages of CD4+ and CD8+ T lymphocytes were higher in the rHBM-S1-N vaccinated birds compared to birds vaccinated with the rHBM-S1 and rHBM-N vaccines. At 5 dpc, the mortality, morbidity and virus re-isolation rate of the birds vaccinated with the rHBM-S1-N vaccine were slightly higher than those vaccinated with the H120 control vaccine but were lower than those vaccinated with the rHBM-S1 and rHBM-N vaccines. The present study demonstrated that the protection of the recombinant baculovirus co-expressing S1 and N proteins was better than that of recombinant baculoviruses mono-expressing the S1 or N protein. Thus, the recombinant baculovirus co-expressing S1 and N proteins could serve as a potential IBV vaccine and this demonstrates that the bivalent subunit vaccine including the S1 and N proteins might be a strategy for the development of an IBV subunit vaccine.

## 1. Introduction

Avian infectious bronchitis (IB) is a highly contagious disease of chickens caused by the infectious bronchitis virus (IBV), belonging to the Gammacoronavirus genus within the Coronaviridae family [1]. IBV affects chickens of all ages and types and primarily infects the respiratory and urogenital systems of chickens, causing massive economic losses [2,3,4]. There are dozens of IBV serotypes related to the extensive variability of the Spike (*S*) protein. There is little or no cross-protection between different serotypes of IBVs or circulating variant viruses, frequently leading to immune failures and making it extremely difficult to control the disease [5,6,7].

Control of the disease currently relies on conventional live-attenuated and inactivated vaccines [8,9,10]. However, live-attenuated vaccines can result in the occurrence of vaccine-like viruses with increased virulence and persistence due to point mutation and recombination with other vaccines or field strains [11,12,13]. Inactivated vaccines are not only high in cost but also do not establish long-term immunity. The application of inactivated vaccines alone has frequently failed to induce strong cellular immunity, resulting in little or no protection [14,15]. Despite the widespread application of these conventional vaccines, IB continues to cause severe economic losses in many countries, underscoring the need to develop new safer and more effective candidate vaccines for the practical control of IBV.

Among new vaccines, subunit vaccines have been showed to elicit strong humoral and cellular immune responses [16,17,18]. Subunit vaccines have significant advantages, such as efficient antigenic presentation, high stability and flexibility in protein or epitope selection, compared with live-attenuated and inactivated vaccines [16]. Additionally, no infectious viral particles are involved in vaccine production, so they are very safe. Subunit vaccines are genetically engineered and are thus easy to mass produce [19]. Subunit vaccines also allow the creation of multivalent vaccines. Thus, subunit vaccines may have the potential to act as safe and effective vaccine candidates.

IBV consists of four structural proteins. They are the spike (S), envelope (E), membrane (M) and nucleocapsid (N) proteins [20]. The S protein is post-translationally cleaved into S1 and S2 subunits [21]. The S1 protein determines the antigenicity and immunogenicity of the virus and plays an important role in the induction of neutralizing and hemagglutination inhibition antibodies, tissue affinity and cell adsorption, all involved in the humoral immune response [22,23,24]. The N protein plays an extremely important role in the replication and assembly of IBV, affecting its immunogenicity and is thus involved in the cellular immune response [25,26,27]. Hence, the S1 and N proteins are the most promising subunit vaccine candidates against IBV. Therefore, the development of subunit vaccines including both the S1 and N proteins has promising prospects.

The baculovirus expression system (BES) is an efficient system for gene expression because of its good safety, high expression and excellent protein processing ability compared to other expression systems. A major advantage of the BES is the ease of scaling up from the laboratory to a large-scale production system [28,29]. In addition, baculoviruses are insect pathogens and thus non-pathogenic for vertebrates. Therefore, the BES is currently widely used for protein expression and vaccine production. There have been a few studies describing the expression of IBV proteins by BES [18,30,31,32,33] but none of these studies have focused on developing and evaluating the immune response of a subunit vaccine from the baculovirus which co-expresses S1 and N proteins of IBV.

This study therefore set out to measure the protective effect of the subunit vaccine co-expressing both the S1 and N proteins of IBV. Moreover, we compared the immune protection offered by this vaccine with those induced by recombinant subunit vaccines expressing S1 protein or N protein alone and H120 inactivated vaccine. The aim is to obtain safer and more effective subunit vaccines, thus providing a novel subunit vaccine candidate for IBV.

## 2. Materials and Methods

### 2.1. Cells, Virus and the Expression Vector

Sf9 insect cells were purchased from Qiyin Biological Technology Co., Ltd. (Jiangyin, China) and cultured in serum-free SF900II medium (GIBCO, Grand Island, NY, USA) at 27 °C. The virulent GX-YL5 strain of IBV was isolated from broilers with nephritis disease and is the representative dominant serotype (different from the H120 strain) isolate in southern China and shared heterologous serotype with the H120 strain [34]. The 50% tracheal organ culture infection dose (TOC-ID_50_) of the IBV GX-YL5 strain was determined as previously described [34]. The euokaryotic expression vector pFastBac^TM^ Dual was the product of Invitrogen (Waltham, MA, USA). 

### 2.2. Gene Cloning and the Recombinant Baculoviruses’ Construction

**Figure 1:**
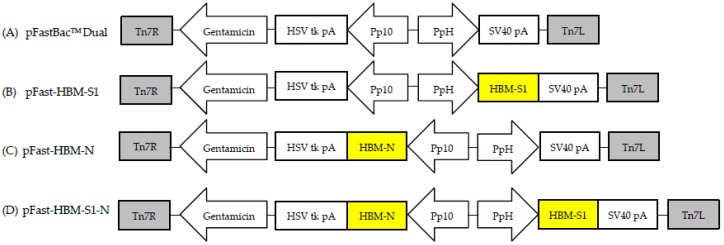
Schematic diagram of *S1*, *N* and *S1-N* genes baculovirus expression systems. (**A**) The schematic diagram of the transfer vector pFastBac^TM^ Dual; (**B**) The *HBM-S1* gene was sub cloned into the vector pFastBac^TM^ Dual under the control of PH promotor; (**C**) The *HBM-N* gene was sub cloned into the vector pFastBac^TM^ Dual under the control of P10 promotor; (**D**) *HBM-S1* and *HBM-N* genes were sub cloned into the vector pFastBacTM Dual under the control of PH and P10 promotors, respectively. The resulting recombinant transposon vectors were named pFast-HBM-S1, pFast-HBM-N and pFast-HBM-S1-N.

In order to increase the expression and yield of heterologous secreted proteins in insect cells, the honeybee melittin (HBM) signal peptide recognized by insect cells was introduced into the BES. The coding mature *S1* (55–1621 bp) (accession number FJ907238.1) and *N* (1–1230 bp) (accession number J548847.1) protein genes of the IBV GX-YL5 strain were amplified by reverse transcription polymerase chain reaction (RT-PCR). The insect signal peptide *HBM* gene was amplified by PCR using a pair of primers with a partial overlapping sequence. Then the fusion genes *HBM-S1* and *HBM-N* were obtained by fusion PCR, cloned into the pEasy-T1 vector and then sub cloned into the transfer vector pFastBac^TM^ Dual (Figure 1A) at the BamH I/Pst I sites as well as the Xho I/Kpn I sites under the control of PH and P10 promotors, respectively (Figure 1B,C) to obtain recombinant transposon vectors pFast-HBM-S1 and pFast-HBM-N. A 6×His tag and a tobacco etch virus (TEV) protease cleavage site were added before the stop codon to facilitate the identification and purification of the expressed proteins. The *HBM-N* gene was then directly sub cloned into pFast-HBM-S1 to yield the recombinant transposon vector pFast-HBM-S1-N. This involved the S1 gene being inserted under the control of promotor PH and the *N* gene being inserted under the control of promoter P10 (Figure 1D). All the recombinant transposon vectors were verified by PCR, restriction endonuclease digestion and sequencing. The verified recombinant transposon vectors were then transformed into DH10Bac^TM^ *Escherichia coli* cells to generate recombinant bacmids rHBM-S1, rHBM-N and rHBM-S1-N, which were identified by PCR with M13 primers. The purified recombinant bacmids rHBM-S1, rHBM-N and rHBM-S1-N were obtained after several instances of screening. They were then transfected into Sf9 insect cells to obtain recombinant baculoviruses through lipofectin-mediated transfection, following the manufacturer's instructions (Invitrogen). The recombinant baculoviruses rHBM-S1, rHBM-N and rHBM-S1-N were identified by PCR with IBV specific primers and M13 primers the recombinant baculovirus titers were determined using end-point dilution analysis. All the primers used in this study are shown in Table S1.

### 2.3. Analysis of Recombinants’ Protein Expression

The expression of recombinant proteins S1, N and S1-N in Sf9 cells were detected using indirect immunofluorescence assay (IFA) and Western blot. For IFA, Sf9 cells were infected with the recombinant baculoviruses rHBM-S1, rHBM-N and rHBM-S1-N at a multiplicity of infection (MOI) of 5, respectively and then fixed with 4% paraformaldehyde at 72 h post-infection. Mouse anti-His IgG (1:2000 dilution, CWBio, Beijing, China) was used as the primary antibody, while fluorescein isothiocyanate (FITC)-labeled goat anti-mouse antibody IgG (1:2000 dilution, CWBio, Beijing, China) was used as the secondary antibody. Specific fluorescent signals in Sf9 cells were observed using fluorescent microscopy.

At 72 h post-infection, cell culture supernatants and cell lysates infected with the recombinant baculoviruses rHBM-S1, rHBM-N and rHBM-S1-N were collected for the analysis of recombinant proteins using Western blot. Protein samples were separated by 12% sodium dodecyl sulfate polyacrylamide gel electrophoresis (SDS-PAGE), transferred onto polyvinylidene fluoride (PVDF) membranes and blocked in 5% skim milk with phosphate buffer solution tween-20 (PBST) buffer. Mouse anti-His IgG (1:2000 dilution, CWBio, Beijing, China) was used as the primary antibody and horseradish peroxidase (HRP)-labeled goat anti-mouse antibody IgG (1:2000 dilution, CWBio, Beijing, China) was used as the secondary antibody. Normal Sf9 cells and Sf9 cells infected with the same amount of wild-type baculovirus were set as negative controls. The proteins were visualized using a diaminobenzidine (DAB) substrate kit as recommended by the supplier.

### 2.4. Immunization of the Birds and the Virus Challenge

The specific-pathogen-free (SPF) chickens used in this study were hatched from fertilized white leghorn SPF eggs (Beijing Merial Vital Laboratory Animal Technology Co., Ltd., Beijing, China) in our facilities and the chickens were housed in separate isolation units until reaching 14 days of age. Fourteen-day-old SPF chickens were assigned randomly into six groups (*n* = 10 chickens/group). Each bird in the rHBM-S1-N, rHBM-S1and rHBM-N groups was injected subcutaneously with 0.5 mL (50 μg) S1-N, S1 and N recombinant proteins, respectively. These proteins were emulsified in Freund’s complete adjuvant on day 14 and boosted with the same dose of proteins emulsified in Freund’s incomplete adjuvant on day 28. Birds in the H120 group, set as a positive control, were injected with the H120 inactivated vaccine. The WT-B group and cell group, serving as negative controls, were set up by replacing the recombinant proteins with emulsified wild-type baculovirus and normal Sf9 cell lysates, respectively. At 14 days post-booster (dpb), all the birds from each group were challenged with 10^5^ TOC-ID_50_ of the IBV GX-YL5 strain in 0.2 mL by the nasal-ocular route. They were monitored daily for clinical signs, morbidity and mortality and they were then euthanized at 5 days post-challenge (dpc). Any birds that died during the observation period were immediately necropsied. Any remaining birds were euthanized at the end of the observation period. The trachea and kidney samples were collected from each bird aseptically for the re-isolation of IBV. These animal experiments were approved by the Animal Care & Welfare Committee of Guangxi University (approval number GXU2016-011, 25th November 2016) and were performed in accordance with animal ethics guidelines and approved protocols.

### 2.5. The Detection of IBV-Specific Antibody

Blood samples were collected from the wing vein of 10 birds in each group prior to prime immunization (0 day) and at 7, 14, 21 and 28 days post-vaccination (dpv) for the detection of the IBV-specific IgG antibody using commercial enzyme-linked immunosorbent assay (ELISA) kits (IDEXX Laboratory, Inc., Westbrook, ME, USA) according to the manufacturer’s recommendations.

### 2.6. Quantification of CD4+ and CD8+ T Lymphocytes

Peripheral blood samples were collected from the wing vein of 10 birds in each group at 0, 7, 14, 21 and 28 dpv. Peripheral blood lymphocytes were isolated and adjusted to 1 × 10^6^ cells/mL, stained with 1 µL Mouse Anti-Chicken CD4-PE and CD8a-FITC antibodies (Wuhan AmyJet Scientific Inc., Wuhan, China) and analyzed for the percentages of CD4+ and CD8+ T lymphocytes by a BD AccuriTM C6 Flow Cytometer (Becton, Dickinson and Company, Minneapolis, MN, USA).

### 2.7. Virus Re-Isolation

All birds were euthanized at 5 dpc. Trachea and kidney mixtures were collected for virus re-isolation through inoculation into 10-day-old SPF chicken embryos via the allantoic cavity route. Allantoic fluid was harvested at 72 h post-inoculation. Three passages in embryonated eggs were conducted. Viral RNA in allantoic fluid was extracted using the EasyPure RNA Purification Kit (TransGen Biotech, Beijing, China) and subjected to a reverse transcriptase polymerase chain reaction targeting the M gene of IBV. The absence of detectable virus in the trachea and kidney was considered to be correlated with the protection of the subunit vaccines against the challenge.

### 2.8. Statistical Analysis

All data were shown as mean values ± standard deviation (mean ± SD) and were compared by a one-way analysis of variance (ANOVA) and Student’s *t*-test using SPSS 18.0 (SPSS Inc., Chicago, IL, USA) biostatistics software. *p*-values less than 0.05 were regarded as significant and those less than 0.01 were regarded as highly significant.

## 3. Results

### 3.1. Confirmation of the Recombinant Baculoviruses

The results of PCR, restriction enzyme digestion analysis (Figure S1) and sequencing showed that recombinant transposon vectors pFast-HBM-S1, pFast-HBM-N and pFast-HBM-S1-N were successfully constructed. The recombinant bacmids rHBM-S1, rHBM-N and rHBM-S1-N were obtained by transforming the verified recombinant transposon vectors to DH10Bac^TM^ *Escherichia coli* cells and identified using PCR. The results of PCR identification showed that the recombinant bacmids rHBM-S1, rHBM-N and rHBM-S1-N were generated (Figure S2). The purified recombinant bacmids rHBM-S1, rHBM-N and rHBM-S1-N were transfected into Sf9 insect cells to obtain the recombinant baculovirus. The recombinant baculovirus rHBM-S1, rHBM-N and rHBM-S1-N were identified using PCR with IBV specific primers and M13 primers. The sizes of the amplified bands matched those of the predicted products (Figure S3).

### 3.2. Identification of the Recombinant Proteins

**Figure 2:**
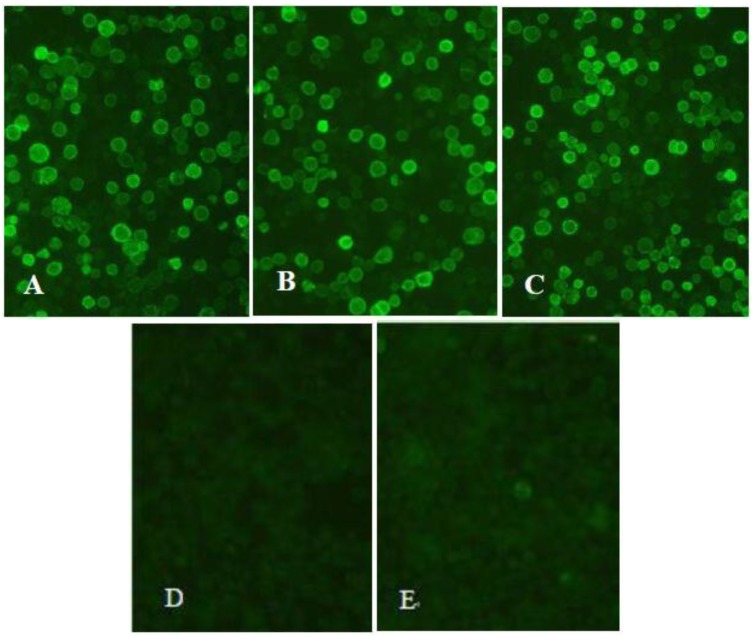
Detection of the recombinant proteins’ expression using indirect immunofluorescence assay (IFA) (200×). Sf9 cells were infected with recombinant baculoviruses at a multiplicity of infection (MOI) of 5 and detected by fluorescence microscopy 72 h post-infection. (**A**) Sf9 cells infected with baculovirus rHBM-S1; (**B**) Sf9 cells infected with baculovirus rHBM-N; (**C**) Sf9 cells infected with baculovirus rHBM-S1-N; (**D**) Sf9 cells infected with wild-type baculovirus; (**E**) Normal Sf9 cells.

**Figure 3:**
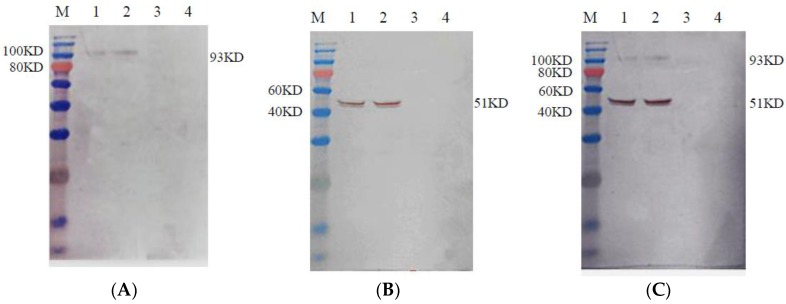
Detection of the recombinant proteins’ expression using Western blot. Sf9 cells were infected with recombinant baculovirus at a MOI of 5. The recombinant proteins were detected 72 h post-infection. (**A**) The recombinant proteins expressed by baculovirus rHBM-S1; (**B**) The recombinant proteins expressed by baculovirus rHBM-N; (**C**) The recombinant proteins expressed by baculovirus rHBM-S1-N. Lane M. Blue Plus IV Protein Marker; Lane 1 culture supernatant; Lane 2 cell lysate; Lane 3 wild-type baculovirus; Lane 4 normal Sf9 cells.

Strong specific immunofluorescence signals were observed in the Sf9 cells infected with the recombinant baculovirus rHBM-S1 (Figure 2A), rHBM-N (Figure 2B) andrHBM-S1-N (Figure 2C) at 72 h post-infection but no fluorescence was detected in the Sf9 cells infected with wild-type baculovirus (Figure 2D) and normal Sf9 cells (Figure 2E). The expected sizes of 93 (Figure 3A) and 51 kDa (Figure 3B) proteins were detected in the culture supernatant and cell lysate infected with rHBM-S1 and rHBM-N, respectively. Two specific bands of 93 and 51 kDa (Figure 3C) proteins were detected in both the culture supernatant and cell lysate infected with rHBM-S1-N. No bands were observed in the culture supernatant or cell lysate infected with wild-type baculovirus and normal Sf9 cells. The results showed that the sizes of expressed recombinant proteins are consistent with the sizes of native S1 or N proteins and these recombinant proteins retained their antigenicity.

### 3.3. Antibody Responses Following the Immunizations

**Figure 4:**
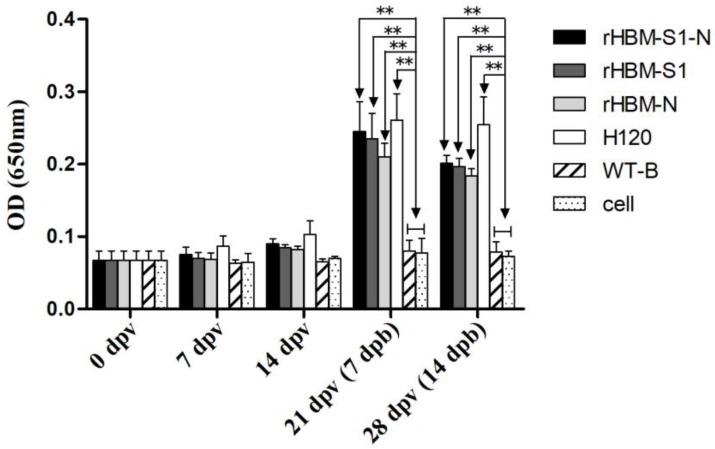
Antibody responses to IBV after vaccination. Serum samples were taken from the six groups of chickens at 0, 7, 14, 21 and 28 days post-vaccination (dpv) and measured by indirect enzyme-linked immunosorbent assay (ELISA). Optical densities were read at 650 nm. Values are expressed as mean values ± standard deviation (mean ± SD) in each group. Statistically significant differences are indicated by * (*p* < 0.05) or ** (*p* < 0.01) (*n* = 10 chickens/group).

At 14 dpv, IBV-specific antibody levels in birds immunized with subunit vaccines rHBM-S1-N, rHBM-S1, rHBM-N and inactivated vaccine strain H120 started to rise (*p* > 0.05). The antibody levels of the vaccinated groups increased notably at 21 and 28 dpv (i.e., 7 and 14 dpb) and were significantly higher than those of the negative control groups (*p* < 0.01) (Figure 4). The highest IBV antibodies in each vaccinated group were observed at 7 dpb. The antibody levels in the rHBM-S1-N group were always higher than those in the rHBM-S1 and rHBM-N groups but the difference was not significant (*p* > 0.05). Similarly, the antibody titers in rHBM-S1 groups were always slightly higher than those in the rHBM-N group (*p* > 0.05). Chickens in the H120 group developed the highest antibody titers throughout the experimental period but the difference was not significant (*p* > 0.05) compared with those in the rHBM-S1-N, rHBM-S1 and rHBM-N groups.

### 3.4. Dynamic Changes of CD4+ and CD8+ T Lymphocytes in Peripheral Blood

**Figure 5:**
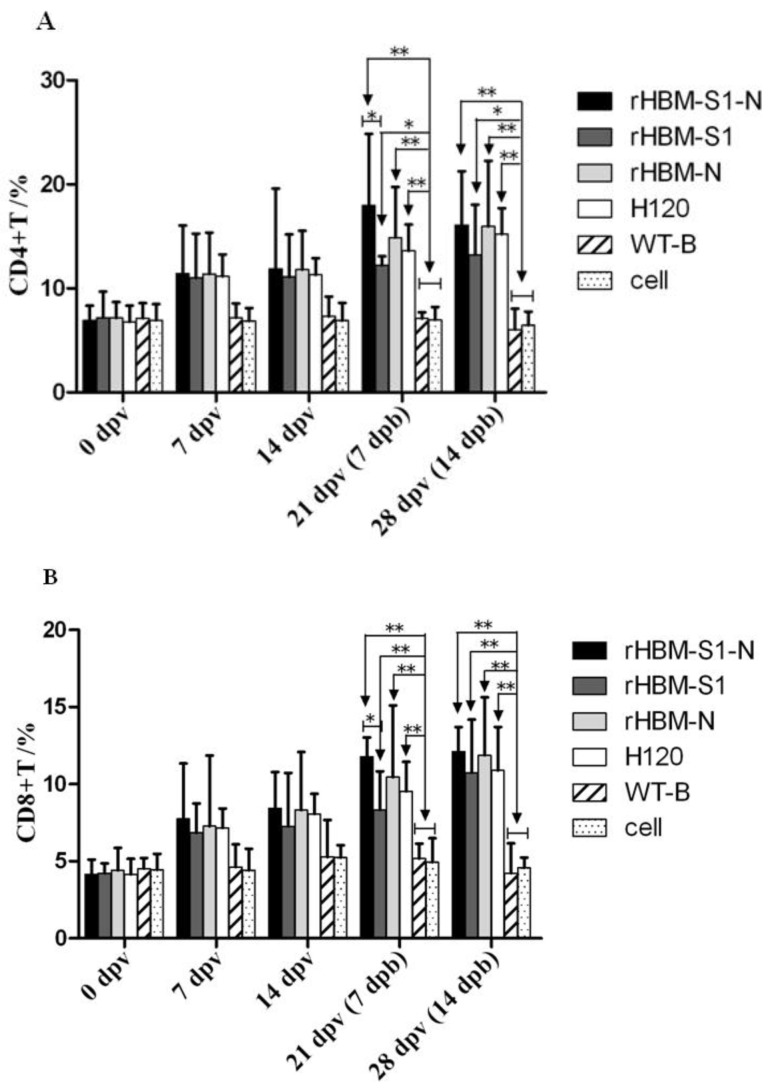
Detection of the percentages of CD4+ and CD8+ T lymphocytes in peripheral blood of vaccinated groups. T lymphocytes were isolated from the six groups of chickens at 0, 7, 14, 21 and 28 dpv. The percentages of (**A**) CD4+ and (**B**) CD8+ T lymphocytes were analyzed by flow cytometry. The data are shown as mean values ± standard deviation (mean ± SD) in each group. Statistically significant differences are indicated by * (*p* < 0.05) or ** (*p* < 0.01) (*n* = 10 chickens/group).

The results showed that the percentages of CD4+ and CD8+ T lymphocytes in vaccinated birds were rising at 7 dpv (Figure 5). At 21 and 28 dpv (i.e., 7 and 14 dpb), the percentages of CD4+ and CD8+ T lymphocytes in the rHBM-S1-N and rHBM-N groups were significantly higher than those of the negative groups (*p* < 0.01). The percentages of CD4+ T lymphocytes in the rHBM-S1 group were significantly higher than those of the negative controls (*p* < 0.05) and the percentages of CD8+ T lymphocytes were significantly higher (*p* < 0.01) compared with the negative controls. Based on percentages of CD4+ and CD8+ T lymphocytes in the vaccinated groups at 21 and 28 dpv (i.e., 7 and 14 dpb), the groups sorted from highest to lowest as follows: the rHBM-S1-N group, the rHBM-N group, the H120 group and the rHBM-S1 group. There was a significant difference between the rHBM-S1-N and rHBM-S1 groups (*p* < 0.05), while no significant difference was noted between other groups (*p* > 0.05).

### 3.5. Protection against the IBV Challenge

At 4 dpc, chickens began to show clinical signs or died. Morbidity, mortality, virus re-isolation rate and protection rate at 5 dpc were summarized in Table 1. Most of the chickens in the two negative control groups (WT-B and cell groups) showed typical signs of IBV infection, such as coughing, sneezing, gasping, tracheal rale and wet droppings. In these groups, there was a 60–70% morbidity rate and a 30–40% mortality rate. In contrast, 20%, 30%, 40% and 10% morbidity rates as well as 0%, 0%, 20% and 0% mortality rates were observed in the rHBM-S1-N, rHBM-S1, rHBM-N and H120 groups, respectively. Birds in the four vaccinated groups exhibited milder clinical signs and gross lesions compared with those in the two negative control groups. In the necropsy at 5 dpc, the kidneys of infected birds were swollen and pale and they exhibited a white sludge indicating urate deposition. Some birds showed trachitis with exudates.

In order to further evaluate the level of protection, the collected trachea and kidney from the challenged birds at 5 dpc were inoculated into 10-day-old SPF chicken embryos for three passages for virus re-isolation. Viral RNA was detected from the infected allantoic fluid of the embryonated eggs using RT-PCR. The virus re-isolation rates for the birds in the rHBM-S1-N, rHBM-S1, rHBM-N and H120 groups were 40%, 50%, 60% and 20% respectively. Those in the WT-B and cell groups had virus re-isolation rates of 80% and 100% respectively (Table 1). Birds in the rHBM-S1-N, rHBM-S1 and rHBM-N groups developed higher levels of protection (i.e., 60%, 50% and 40%, respectively) than those in the WT-B and cell groups (i.e., 20% and 0%, respectively) but lower levels of protection than that in the H120 group (80%). No protection was observed in the cell group and a low level of protection (20%) was observed in the WT-B group. 

## 4. Discussion

IB is a highly contagious and acute viral disease of chickens which causes massive economic losses. Live-attenuated and inactivated vaccines are commonly used to control this disease. However, live-attenuated vaccines may lead to the occurrence of new serotypes or variants of IBV due to mutation and recombination [11,12,13]. Also, inactivated vaccines often fail to induce strong cellular immunity and have the disadvantage of high manufacturing costs [14,15]. Hence, there is an urgent need to develop new, safe and effective vaccines to control the disease. Recombinant subunit vaccines have been shown to elicit strong humoral and cellular immune responses and are very safe [16,17,18]. Therefore, subunit vaccines may serve as potential vaccine candidates in the future.

In the current study, significantly higher antibody levels, higher percentages of CD4+ and CD8+ T lymphocytes and higher protection rates were demonstrated when using the subunit vaccines rHBM-S1-N, rHBM-S1 and rHBM-N compared to the WT-B and cells at 7 and 14 dpb. Therefore, the recombinant subunit vaccines rHBM-S1-N, rHBM-S1 and rHBM-N could induce the immunized chickens to produce humoral and cellular immunity to resist and eliminate the IBV infection, indicating that the recombinant bacculovirus vaccines expressing the S1 and N proteins of IBV may be effective subunit vaccines for future use.

The immune efficacy of subunit vaccine rHBM-S1 containing the S1 protein was stronger than that of the subunit vaccine rHBM-N containing the N protein in the generation of humoral immune responses. However, the immune efficacy of subunit vaccine rHBM-N was stronger than that of rHBM-S1 in the generation of cellular immune responses. The results confirmed that the S1 protein and N protein play a major role in humoral and cellular immune responses, respectively, which is in agreement with the previous investigations [23,26,35]. The immune efficacy of subunit vaccine rHBM-S1-N was better than that of subunit vaccines rHBM-S1 or rHBM-N in generating humoral and cellular immune responses. The reason for this was that rHBM-S1-N expressed both the S1 and N proteins of IBV and stimulated both the humoral and cellular immune responses simultaneously. Therefore, the subunit vaccine rHBM-S1-N performed better in the activation of virus-specific immune responses. Although the S1 subunit is the major inducer of neutralizing antibodies, vaccination with the S1 protein did not confer adequate protection against challenge [17]. A previous study even found that the antigenicity of the N protein is better than that of the S1 protein [36]. In another study, the cell-mediated immune response induced by the N protein was higher than that induced by either the S1 protein or H120 [37]. These results and ours indicate the importance of generating a subunit vaccine that contains both the *S1* and the *N* genes.

A previous study showed that the BacMam virus Ac-CMV-S1, which expresses the S1 glycoprotein of IBV-M41, was deficient in the induction of IBV-specific antibody compared with that induced by the inactivated vaccine [18]. In our study, the antibody levels in the groups rHBM-S1-N, rHBM-S1 and rHBM-N were slightly lower than that of the H120 group at 7 and 14 dpb, which agreed with the previous study [18]. A possible reason for this was that the H120 vaccine was derived from the whole virus and was comprised of almost all the epitopes of IBV. Another reason may be related to the fact that the coated antigen of the ELISA kit used in the study was the Mass-type virus strain. Based on the percentages of CD4+ and CD8+ T lymphocytes, the groups ordered from highest to lowest are the rHBM-S1-N group, rHBM-N group, H120 group and rHBM-S1 group. It was surprising that the cellular immune response in the rHBM-N group was higher than that in the H120 group, although the difference was not found to be statistically significant. A previous study showed that Ac-CMV-S1 induced a significantly better cellular immune response in SPF chickens compared with that induced by the inactivated vaccine [18], which was consistent with our results. The cell-mediated immune response induced by rHBM-N was higher than that induced by either H120 or rHBM-S1. One possible explanation was a more efficient presentation of N peptides by antigen-presenting cells following effective uptake, processing, or presentation on MHC receptors of these cells [37]. Therefore, the necessity of co-expressing the S1 and N proteins is again stressed.

In the present study, chickens in the H120 group did not obtain 100% protection against the IBV GX-YL5 strain challenge. This was possibly because the strain used as the H120 vaccine was heterologous to the GX-YL5 strain which was used as the challenge strain [34]. This was consistent with previous studies [38,39,40]. Another reason could be the absence of live priming for the inactivated H120 vaccine [14,41]. In addition, the protection rates of subunit vaccines rHBM-S1-N (60%), rHBM-S1 (50%) and rHBM-N (40%) were lower than that of the traditional vaccine H120 (80%). A previous description reported 55% protection against the virulent M41 challenge from the constructed baculovirus expressing the S1 protein of the IBV M41 strain, which was lower than that for the inactivated vaccine (72%) [42]. Only 50% protection was obtained against the homologous KM91 strain challenge after three immunizations with the IBV S1 glycoprotein expressed by a recombinant baculovirus [32]. A similar result was shown in another study [43].

It was surprising that the protection rate of the rHBM-S1-N vaccine (60%) against the homologous GX-YL5 strain challenge was lower than that of the H120 vaccine (80%) against the heterologous GX-YL5 strain challenge. There are some possible reasons for this. Firstly, the rHBM-S1-N subunit vaccines contained only part of the whole immunogenicity of the virus. This could not lead to an immune effect that was as good as the whole-virus vaccine H120. Secondly, the cell lysates were used to prepare the oil emulsion vaccines for the chickens’ immunizations and it is unknown whether the complicated composition of the cell lysate had an effect on the immunized birds. Thirdly, the immune dose of 50 μg/bird may be not enough. Doses of 150 μg/bird were used to immunize chickens in other studies [37,44]. Finally, two immunizations may be not enough to elicit an effective immune response. A previous study pointed out that at least four immunizations with purified S1 glycoprotein were needed to induce protection against the homologous N1/62 strain challenge [45]. Similarly, three immunizations were performed and only 50% protection was obtained against the homologous IBV in the previous study [32]. In this study, we only immunized the chickens twice with the recombinant proteins; this may be a reason for the low protection conferred by the subunit vaccines.

Live attenuated vaccines are applied from day-old to achieve early protection and are boosted with the inactivated vaccines in the case of future layers and breeders. Although the subunit vaccine rHBM-S1-N could not provide complete protection against IBV infection, it conferred higher protection than rHBM-S1 and rHBM-N vaccines. Thus, it still can be used as an alternative vaccine for boosting the primary vaccination with traditional vaccine/vaccines against this disease. As we know, IBV continuously evolves and there are scores of serotypes. Little or no cross-protection confers between different serotypes of IBVs [6,7]. Therefore, universal IBV vaccines that protect against varying serotypes of IBV are imperative. Our results should enable the generation of multivalent vaccines to prevent more serotypes of IBV, especially the newly emerging virus strains. The vaccine rHBM-S1-N can also be applied by combining it with traditional vaccines to reduce the occurrence of variants caused by virus mutations and recombination. Therefore, the subunit vaccine rHBM-S1-N is an alternative to the traditional IBV vaccine. Our study also provides reference and ideas for the development of subsequent new vaccines. Further study is needed to determine the effectiveness of combining the subunit vaccines with traditional vaccines.

In this study, a 20% protection rate was conferred following IBV challenge in the wild-type baculoviruses control group and the protection rate of the wild-type baculoviruses control group was slightly higher than that of the cell control group, which was consistent with previous reports [18,41]. A possible reason could be that the baculovirus induced innate immunity through the Toll-like receptor 9 and MyD88-dependent signaling pathway and stimulates the production of various inflammatory cytokines [46]. Therefore, vaccination with baculovirus alone may result in non-specific immunity, which could provide only a small amount of protection against an IBV challenge.

It is critical to obtain high-level secreted expressions of recombinant proteins with native activities. HBM is an insect-derived signal peptide. It has been reported that the introduction of the HBM signal peptide could increase the expression of foreign proteins, enhance the activity of expressed proteins and achieve the secretion of foreign proteins in the baculovirus system [47,48,49]. There have been a few studies about expressing IBV proteins using BES [18,30,31,32,33] but none of these studies have focused on introduction of the HBM signal peptide into BES to express IBV proteins. Therefore, the HBM signal peptide was introduced in the present study. The results showed that the recombinant proteins S1-N, S1 and N could be expressed both in the culture supernatant and cell lysate. Thus, the introduction of the HBM signal peptide into BES can be applied to express other proteins from other pathogens.

In summary, the current study showed that the constructed recombinant baculoviruses could elicit both cellular and humoral immune responses to a certain degree and the protection offered by the recombinant baculovirus co-expressing S1 and N proteins was better than that of recombinant baculoviruses containing the S1 or N protein alone. To our knowledge, this is the first report that proved that bivalent subunit vaccines, including S1 and N proteins of IBV, could induce higher immune responses and provide greater protection against infection. The recombinant baculovirus co-expressing S1 and N proteins could serve as a potential IBV vaccine and a bivalent subunit vaccine including the S1 and N proteins might be a strategy for the development of an IBV subunit vaccine.

## Figures and Tables

**Table 1:** Protective efficacy of recombinant subunit vaccines against the challenge of the virulent GX-YL5 strain of infectious bronchitis virus.

Groups	Morbidity (%) ^a^	Mortality (%) ^b^	Virus Re-Isolation Rate (%) ^c^	Protection Rate (%) ^d^
rHBM-S1-N	20 (2/10)	0 (0/10)	40 (4/10)	60 (6/10)
rHBM-S1	30 (3/10)	0 (0/10)	50 (5/10)	50 (5/10)
rHBM-N	40 (4/10)	20 (2/10)	60 (6/10)	40 (4/10)
H120	10 (1/10)	0 (0/10)	20 (2/10)	80 (8/10)
WT-B	60 (6/10)	30 (3/10)	80 (8/10)	20 (2/10)
Cell	70 (7/10)	40 (4/10)	100 (10/10)	0 (0/10)

^a^ Morbidity was determined by dividing the number of affected chickens by the total number of chickens in each group; ^b^ Mortality was determined by dividing the number of dead chickens by the total number of chickens in each group; ^c^ The virus re-isolation rate was determined by dividing the number of chickens with virus re-isolation from the tracheas and kidneys of challenged chickens by the total number of chickens in each group; ^d^ The protection rate was determined by dividing the number of unaffected chickens (without detection in trachea and kidney) by the total number of chickens in each group.

## Supplementary Materials

The following are available online at http://www.mdpi.com/1999-4915/10/7/347/s1, Figure S1: Identification of recombinant transposon vectors pFast-HBM-S1, pFast-HBM-N and pFast-HBM-S1-N by PCR and restriction endonuclease digestion. Figure S2: Identification of recombinant bacmids rHBM-S1, rHBM-N and rHBM-S1-N by PCR with M13 primers. Figure S3: Identification of recombinant baculovirus by PCR with IBV specific primers and M13 primers. Table S1: All the primers used in this study.

## References

[1] De Wit J.J., Cook J.K., van der Heijden H.M. (2011). Infectious bronchitis virus variants: A review of the history, current situation and control measures. Avian Pathol..

[2] Cavanagh D. (2007). Coronavirus avian infectious bronchitis virus. Vet. Res..

[3] De Wit J.J., Swart W.A., Fabri T.H. (2010). Efficacy of infectious bronchitis virus vaccinations in the field: Association between the alpha-IBV IgM response, protection and vaccine application parameters. Avian Pathol..

[4] Jackwood M.W. (2012). Review of infectious bronchitis virus around the world. Avian Dis..

[5] Dhama K., Singh S.D., Barathidasan R., Desingu P.A., Chakraborty S., Tiwari R., Kumar M.A. (2014). Emergence of Avian Infectious Bronchitis Virus and its variants need better diagnosis, prevention and control strategies: A global perspective. Pak. J. Biol. Sci..

[6] Huo Y.F., Huang Q.H., Lu M., Wu J.Q., Lin S.Q., Zhu F.Z., Zhang X.M., Huang Y.Y., Yang S.H., Xu C.T. (2016). Attenuation mechanism of virulent infectious bronchitis virus strain with QX genotype by continuous passage in chicken embryos. Vaccine.

[7] Chen L.F., Zhang T.T., Han Z.X., Liang S.L., Xu Y., Xu Q.Q., Chen Y.Q., Zhao Y., Shao Y.H., Li H.X. (2015). Molecular and antigenic characteristics of Massachusetts genotype infectious bronchitis coronavirus in China. Vet. Microbiol..

[8] Cavanagh D. (2003). Severe acute respiratory syndrome vaccine development: Experiences of vaccination against avian infectious bronchitis coronavirus. Avian Pathol..

[9] Tang M.J., Wang H.N., Zhou S., Tian G.B. (2008). Enhancement of the immunogenicity of an infectious bronchitis virus DNA vaccine by a bicistronic plasmid encoding nucleocapsid protein and interleukin-2. J. Virol. Methods.

[10] Tarpey I., Orbell S.J., Britton P., Casais R., Hodgson T., Lin F., Hogan E., Cavanagh D. (2006). Safety and efficacy of an infectious bronchitis virus used for chicken embryo vaccination. Vaccine.

[11] Van V.S., Toro H. (2008). Rapid selection in chickens of subpopulations within ArkDPI-derived infectious bronchitis virus vaccines. Avian Pathol..

[12] McKinley E.T., Deborah A.H., Jackwood M.W. (2008). Avian coronavirus infectious bronchitis attenuated live vaccines undergo selection of subpopulations and mutations following vaccination. Vaccine.

[13] Shi X.M., Zhao Y., Gao H.B., Jing Z., Wang M., Cui H.Y., Tong G.Z., Wang Y.F. (2011). Evaluation of recombinant fowlpox virus expressing infectious bronchitis virus S1 gene and chicken interferon- gamma gene for immune protection against heterologous strains. Vaccine.

[14] Ladman B.S., Pope C.R., Ziegler A.F., Swieczkowski T., Callahan C.J., Davison S., Gelb J. (2002). Protection of chickens after live and inactivated virus vaccination against challenge with nephropathogenic infectious bronchitis virus PA/Wolgemuth/98. Avian Dis..

[15] Meeusen E.N., Walker J., Peters A., Pastoret P.P., Jungersen G. (2007). Current status of veterinary vaccines. Clin. Microbiol. Rev..

[16] Yang T., Wang H.N., Wang X., Tang J.N., Lu D., Zhang Y.F., Guo Z.C., Li Y.L., Gao R., Kang R.M. (2009). The protective immune response against infectious bronchitis virus induced by multi-epitope based peptide vaccines. Biosci. Biotechnol. Biochem..

[17] Eldemery F., Joiner K.S., Toro H., Van V.S. (2017). Protection against infectious bronchitis virus by spike ectodomain subunit vaccine. Vaccine.

[18] Zhang J., Chen X.W., Tong T.Z., Ye Y., Liao M., Fan H.Y. (2014). BacMam virus-based surface display of the infectious bronchitis virus (IBV) S1 glycoprotein confers strong protection against virulent IBV challenge in chickens. Vaccine.

[19] Bande F., Arshad S.S., Bejo M.H., Moeini H., Omar A.R. (2015). Progress and challenges toward the development of vaccines against avian infectious bronchitis. J. Immunol. Res..

[20] Mo M.L., Li M., Huang B.C., Fan W.S., Wei P., Wei T.C., Cheng Q.Y., Wei Z.J., Lang Y.H. (2013). Molecular Characterization of Major Structural Protein Genes of Avian Coronavirus Infectious Bronchitis Virus Isolates in Southern China. Viruses.

[21] Cavanagh D., Davis P.J., Cook J.K., Li D., Kant A., Koch G. (1992). Location of the amino acid differences in the S1 spike glycoprotein subunit of closely related serotypes of infectious bronchitis virus. Avian Pathol..

[22] Cavanagh D., Davis P.J., Darbyshire J.H., Peters R.W. (1986). Coronavirus IBV: Virus retaining spike glycopolypeptide S2 but not S1 is unable to induce virus-neutralizing or haemagglutination-inhibiting antibody, or induce chicken tracheal protection. J. Gen. Virol..

[23] Moore K.M., Jackwood M.W., Hilt D.A. (1997). Identification of amino acids involved in a serotype and neutralization specific epitope within the s1 subunit of avian infectious bronchitis virus. Arch. Virol..

[24] Wickramasinghe I.N., van Beurden S.J., Weerts E.A., Verheije M.H. (2014). The avian coronavirus spike protein. Virus Res..

[25] Seo S.H., Wang L., Smith R., Collisson E.W. (1997). The carboxyl-terminal 120-residue polypeptide of infectious bronchitis virus nucleocapsid induces cytotoxic T lymphocytes and protects chickens from acute infection. J. Virol..

[26] Yu D., Han Z.X., Xu J., Shao Y.H., Li H.X., Kong X.G., Liu S.W. (2010). A novel B-cell epitope of avian infectious bronchitis virus N protein. Virol. Immunol..

[27] Kim T.W., Lee J.H., Hung C.F., Peng S., Roden R., Wang M.C., Viscidi R., Tsai Y.C., He L.M., Chen P.J. (2004). Generation and characterization of DNA vaccines targeting the nucleocapsid protein of severe acute respiratory syndrome coronavirus. J. Virol..

[28] Ikonomou L., Schneider Y.J., Agathos S.N. (2003). Insect cell culture for industrial production of recombinant proteins. Appl. Microbiol. Biotechnol..

[29] Bahia D., Cheung R., Buchs M., Geisse S., Hunt I. (2005). Optimisation of insect cell growth in deep-well blocks: Development of a high-throughput insect cell expression screen. Protein Expr. Purif..

[30] Liu G.M., Lv L.S., Yin L.J., Li X.M., Luo D.G., Liu K., Xue C.Y., Cao Y.C. (2013). Assembly and immunogenicity of coronavirus-like particles carrying infectious bronchitis virus M and S proteins. Vaccine.

[31] Abdel-Moneim A.S., Giesow K., Keil G.M. (2014). High-level protein expression following single and dual gene cloning of infectious bronchitis virus *N* and *S* genes using baculovirus systems. Viral Immunol..

[32] Song C.S., Lee Y.J., Lee C.W., Sung H.W., Kim J.H., Mo I.P., Izumiya Y., Jang H.K., Mikami T. (1998). Induction of protective immunity in chickens vaccinated with infectious bronchitis virus S1 glycoprotein expressed by a recombinant baculovirus. J. Gen. Virol..

[33] Xu P.W., Wu X., Wang H.N., Ma B.C., Ding M.D., Yang X. (2016). Assembly and immunogenicity of baculovirus-derived infectious bronchitis virus–like particles carrying membrane, envelope and the recombinant spike proteins. Biotechnol. Lett..

[34] Li M., Wang X.Y., Wei P., Chen Q.Y., Wei Z.J., Mo M.L. (2012). Serotype and genotype diversity of infectious bronchitis viruses isolated during 1985-2008 in Guangxi, China. Arch. Virol..

[35] Ignjatovic J., Sapats S. (2005). Identification of previously unknown antigenic epitopes on the S and N proteins of avian infectious bronchitis virus. Arch. Virol..

[36] Ignjatovic J., Galli U. (1995). Immune responses to structural proteins of avian infectious bronchitis virus. Avian Pathol..

[37] Meir R., Krispel S., Simanov L., Eliahu D., Maharat O., Pitcovski J. (2012). Immune responses to mucosal vaccination by the recombinant S1 and N proteins of infectious bronchitis virus. Viral Immunol..

[38] Liu S.W., Chen J.F., Han Z.X., Zhang Q.X., Shao Y.H., Kong X.G., Tong G.Z. (2006). Infectious bronchitis virus: *S1* gene characteristics of vaccines used in China and efficacy of vaccination against heterologous strains from China. Avian Pathol..

[39] Liu S.W., Zhang X.N., Wang Y., Li C.R., Liu Q.R., Han Z.X., Zhang Q.X., Kong X.G., Tong G.Z. (2009). Evaluation of the protection conferred by commercial vaccines and attenuated heterologous isolates in China against the CK/CH/LDL/97I strain of infectious bronchitis coronavirus. Vet. J..

[40] Zhang X.N., Wang Y., Li C.R., Liu Q.R., Han Z., Shao Y.H., Liu S.W., Kong X.G. (2008). Evaluation of the protection conferred by several avian infectious bronchitis attenuated vaccines against the field strain CK/CH/LDL/97 I in China. Bing Du Xue Bao.

[41] Abe T., Takahashi H., Hamazaki H., Miyano-Kurosaki N., Matsuura Y., Takaku H. (2003). Baculovirus induces an innate immune response and confers protection from lethal influenzavirus infection in mice. J. Immunol..

[42] Fan H.Y., Luo Q., Chen X.W., Ye Y., Xin C.A., Liao M. (2013). Immunogenicity of a recombinant pseudotype baculovirus expressing the S1 protein of infectious bronchitis virus in specific pathogen free (SPF) chickens. Acta Vet. Zootech. Sin..

[43] Dai Y.B., Ding C., Liu M., Chen D.S., Pan J.Y., Lu Y.H., Chen P.Y., Cai B.X. (2003). Immunogenicity evaluation of S1 glycoproteins from infectious bronchitis virus isolates Js/95/03 and SD/97/01 expressed by recombinant baculoviruses. Chin. J. Vet. Sci..

[44] Tan L., Zhang Y.Q., Liu F., Yuan Y.M., Zhan Y.J., Sun Y.J., Qiu X.S., Meng C.C., Song C.P., Ding C. (2016). Infectious bronchitis virus poly-epitope-based vaccine protects chickens from acute infection. Vaccine.

[45] Ignjatovic J., Galli L. (1994). The S1 glycoprotein but not the N or M proteins of avian infectious bronchitis virus induces protection in vaccinated chickens. Arch. Virol..

[46] Abe T., Hemmi H., Miyamoto H., Moriishi K., Tamura S., Takaku H., Akira S., Matsuura Y. (2005). Involvement of the Toll-like receptor 9 signaling pathway in the induction of innate immunity by baculovirus. J. Virol..

[47] Vihko P., Kurkela R., Porvari K., Herrala A., Lindfors A., Lindqvist Y., Schneider G. (1993). Rat acid phosphatase: Overexpression of active, secreted enzyme by recombinant baculovirus-infected insect cells, molecular properties andcrystallization. Proc. Natl. Acad. Sci. USA.

[48] Wang Z.M., Tong L.L., Grant D., Cihlar T. (2001). Expression and characterization of soluble human parainfluenza virus type 1 hemagglutinin-neuraminidase glycoprotein. J. Virol. Methods.

[49] Van der Geld Y.M., Smook M., Huitema M.G., Harmsen M.C., Limburg P.C., Kallenberg C. (2002). Expression of recombinant proteinase 3, the autoantigen in Wegener’s granulomatosis, in insect cells. J. Immunol. Methods.

