# Supplementary material for: Protection against Virulent Infectious Bronchitis Virus Challenge Conferred by a Recombinant Baculovirus Co-Expressing S1 and N Proteins
Source: Viruses. 2018 Jun 27;10(7):347. doi: 10.3390/v10070347 (PMC6071288; doi:10.3390/v10070347)
Supplement: Supplementary file 1 [file viruses-10-00347-s001.pdf]

Supplementary Figure Captions

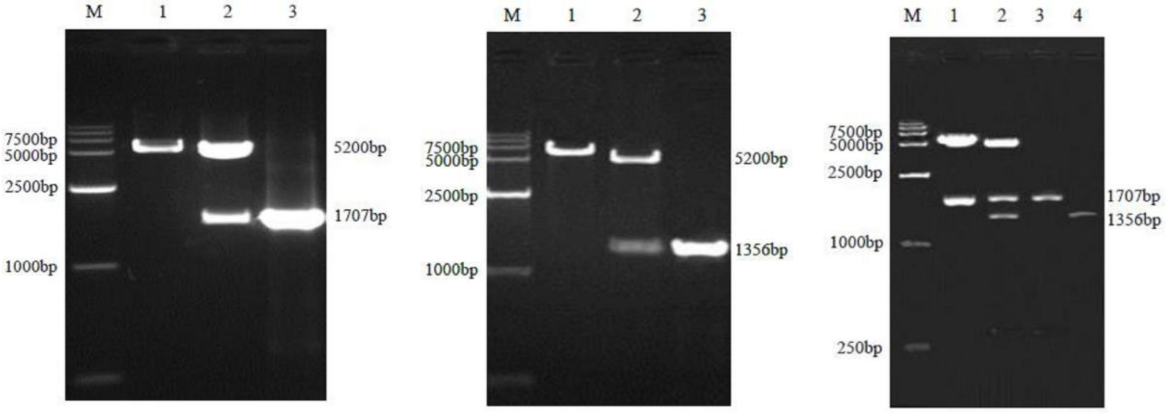

(A) **(B)** **(C)**

Figure S1. Identification of recombinant transposon vectors pFast-HBM-S1, pFast-HBM-N and pFast-HBM-S1-N by PCR and restriction endonuclease digestion. **(A)** Identification of recombinant transposon vectors pFast-HBM-S1. Lane M. DL15000 DNA Marker; Lane 1. pFast-HBM-S1 was digested with *Pst* Ⅰ, Lane 2. pFast-HBM-S1 digested with *Bam*H Ⅰ and *Pst* Ⅰ. Lane 3. PCR identification of pFast-HBM-S1 with HBM-S1 specific primers. **(B)** Identification of recombinant transposon vector pFast-HBM-N. Lane M. DL15000 DNA Marker; Lane 1. pFast-HBM-N was digested with *Kpn* Ⅰ. Lane 2. pFast-HBM-N was digested with *Xho* Ⅰ and *Kpn* Ⅰ. Lane 3. PCR identification of pFast-HBM-N with HBM-N specific primers. **(C)** Identification of recombinant transposon vector pFast-HBM-S1-N. Lane M. DL15000 DNA Marker; Lane 1. pFast-HBM-S1-N was digested with *Bam*H Ⅰ and *Pst*, Lane 2. pFast-HBM-S1-N was digested with *Bam*H Ⅰ, *Pst* Ⅰ, *Xho* Ⅰ and *Kpn* Ⅰ. Lane 3. PCR identification of pFast-HBM-S1-N with HBM-S1 specific primers. Lane 4. PCR identification of pFast-HBM-S1-N with HBM-N specific primers.

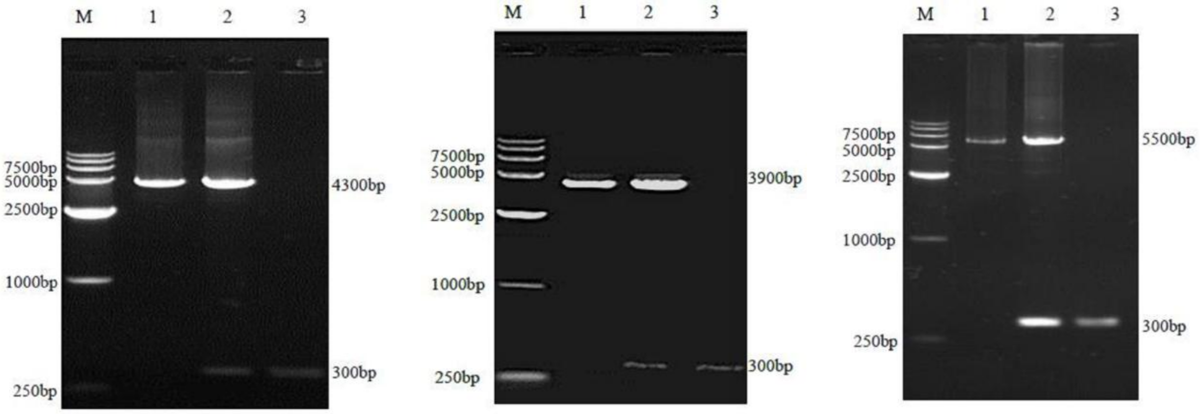

(**A**) (**B**) (**C**)

Figure S2. Identification of recombinant bacmids rHBM-S1, rHBM-N and rHBM-S1-N by PCR with M13 primers. **(A)** Identification of recombinant bacmids rHBM-S1. Lane M. DL15000 DNA Marker; Lane 1. PCR product of the purified bacmids rHBM-S1; Lane 2. PCR product of the unpurified bacmids rHBM-S1; Lane 3. PCR product of the wild bacmid. **(B)** Identification of recombinant bacmids rHBM-N. Lane M. DL15000 DNA Marker; Lane 1. PCR product of the purified bacmids rHBM-N; Lane 2. PCR product of the unpurified bacmids rHBM-N; Lane 3. PCR product of the wild bacmid. **(C)** Identification of recombinant bacmids rHBM-S1-N. Lane M. DL15000 DNA Marker; Lane 1. PCR product of the purified bacmids rHBM-S1-N; Lane 2. PCR product of the unpurified bacmids rHBM-S1-N; Lane 3. PCR product of the wild bacmid.

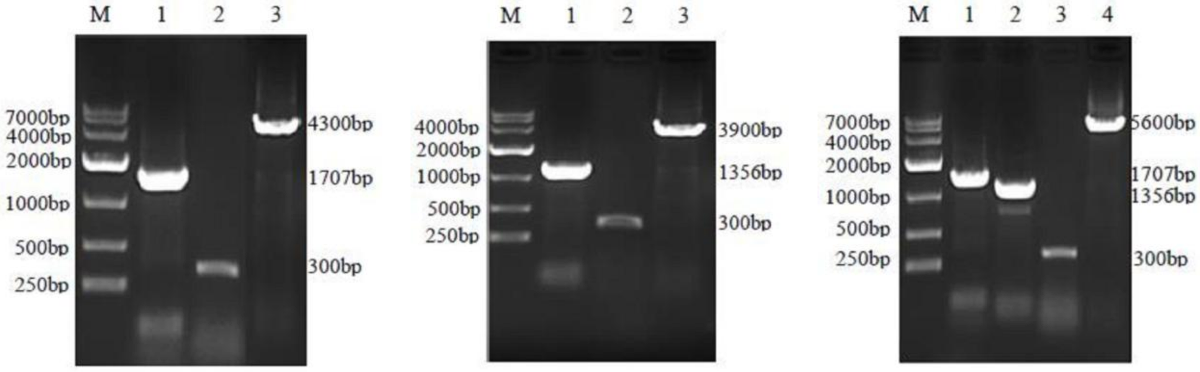

(**A**) (**B**) (**C**)

Figure S3. Identification of recombinant baculovirus by PCR with IBV specific primers and M13 primers. **(A)** Identification of recombinant baculovirus rHBM-S1. Lane M. DL10000 DNA Maker;Lane 1, PCR product of baculovirus rHBM-S1 with S1 gene specific primers; Lane 2, PCR product of wild baculovirus with M13 primers; Lane 3, PCR product of baculovirus rHBM-S1 with M13 primers. **(B)** Identification of recombinant baculovirus rHBM-N. Lane M. DL10000 DNA Maker; Lane 1, PCR product of baculovirus rHBM-N with N gene specific primers; Lane 2, PCR product of wild baculovirus with M13 primers; Lane 3, PCR product of baculovirus rHBM-N with M13 primers. **(C)** Identification of recombinant baculovirus rHBM-S1-N. Lane M. DL10000 DNA Maker; Lane 1, PCR product of baculovirus rHBM-S1-N with S1 gene specific primers; Lane 2. PCR product of baculovirus rHBM-S1-N with N gene specific primers; Lane 3, PCR product of wild baculovirus with M13 primers; Lane 4, PCR product of baculovirus rHBM-S1-N with M13 primers.

Table S1. All the primers used in this study

Primer ID	Sequence(5′→3′)	Gene size
HBM-F1	CGC***GGATCC***ATGAAATTCTTAGTCAACGTTGCCCTTGTTTTTATGGTC (*Bam*H Ⅰ)	78 bp
HBM-R1	TCGATCCGCATAGATGTAAGAAATGTATACGACCATAAAAACAAGGGC	
S1-F	CGC***GGATCC***ATGTTGGGGAAGTCACTGTTTTTAGTG (*Bam*H Ⅰ)	1692 bp
S1-R	CCC***AAGCTT***TCAGTGATGGTGATGGTGATGGCCTTGAAAGTACAAGTTTTCACGTCTGTGACGACGACTTTCAT (*Hin*d Ⅲ)	
HBM-S1-F	CATTTCTTACATCTATGCGGATCGAAATTTGTTTCATTCTGGT	1707 bp
HBM-S1-R	CAAA***CTGCAG***TCAGTGATGGTGATGGTGATGGCCTTGAAAGTACAAGTTTTCACGTCTGTGACGACGACTTTC (*Pst* Ⅰ)	
HBM-F2	CCG***CTCGAG***ATGAAATTCTTAGTCAACGTTGCCCTTGTTTTTATGGTC (*Xho* Ⅰ)	78 bp
HBM-R2	TCGATCCGCATAGATGTAAGAAATGTATACGACCATAAAAACAAGGGC	
N-F	CATTTCTTACATCTATGCGGATCGAATGGCAAGCGGTAAAGCAGCT	1303 bp
HBM-N-R	CGG***GGTACC***TCAGTGATGGTGATGGTGATGGCCTTGAAAGTACAAGTTTTCAAGTTCATTCTCTCCAAGTGCTGAATCG (*Kpn* Ⅰ)	
HBM-N-F	CCG***CTCGAG***ATGAAATTCTTAGTCAACGTTGCCCTTGTTTTTATGGTCGTATACATTTCTTACATCTATGCGGATCGAATGGCAAGCGGTAAAGCAGCT (*Xho* Ⅰ)	1356 bp
HBM-N-R	CGG***GGTACC***TCAGTGATGGTGATGGTGATGGCCTTGAAAGTACAAGTTTTCAAGTTCATTCTCTCCAAGTGCTGAATCG (*Kpn* Ⅰ)	
M13-F	CCCAGTCACGACGTTGTAAAACG	≈2560 bp + size of insert
M13-R	AGCGGATAACAATTTCACACAGG	
M-F	GGTAGAAAACTTAACAATCC	740 bp
M-R	AAGACTACTTCCTCCTGTTG	

Note: The italicized is the restriction enzyme site, the underlined are the 6 × His tag and the TEV protease cleavage site added downstream of the target gene
